# Relative Prevalence and Associated Factors of Complex Regional Pain Syndrome Type I in Patients with Radial Head Fractures Treated with Open Reduction and Internal Fixation: A Cross-Sectional Study

**DOI:** 10.1155/2022/9214404

**Published:** 2022-05-19

**Authors:** Siming Jia, Chuan Ren, Xiaoying Shi, Tailong Shi, Dacheng Sun, Yuqin Zhang, Kai Ding, Hao Du, Yanbin Zhu, Wei Chen

**Affiliations:** ^1^Department of Orthopedic Surgery, The Third Hospital of Hebei Medical University, No. 139 Ziqiang Road, Shijiazhuang 050051, China; ^2^NHC Key Laboratory of Intelligent Orthopedic Equipment, The Third Hospital of Hebei Medical University, Shijiazhuang, China; ^3^Key Laboratory of Biomechanics of Hebei Province, Shijiazhuang, Hebei, China; ^4^Department of Neurology, Hebei General Hospital, Shijiazhuang 050011, Hebei, China; ^5^The First Affiliated Hospital, College of Clinical Medicine of Henan University of Science and Technology, Luoyang 471003, China

## Abstract

**Objective:**

This *cross-sectional* study aimed to examine the incidence and associated factors of complex regional pain syndrome type I (CRPS I) in patients who underwent open reduction and internal fixation (ORIF) for radial head fractures.

**Methods:**

The study enrolled 601 radial head fracture patients treated with ORIF, 523 of which completed the 1-year follow-up. The incidence of CRPS I in those patients was assessed using the Budapest criteria. Patients were then divided into 2 groups: patients with CRPS I (*n* = 28) and patients without CRPS I (*n* = 495). The patients' demographic and clinical data before the operation were prospectively collected by our team. Independent *t*-tests and *χ*^2^ tests were used as univariate analyses to compare the demographic and clinical data between the two groups. Meanwhile, multivariate regression analysis was conducted to identify the associated risk factors for CRPS I.

**Results:**

The incidence of CRPS I in patients with radial head fractures treated with ORIF was 5.5% during the first year following surgery. Significant differences were observed in age, gender, type of trauma, modified Mason Classification, and depressive personality disorders. The logistic regression analysis revealed that the female gender, modified Mason type III fractures, and depressive patients were significantly more likely to develop CRPS I (*p*=0.021, 0.023, and 0.025, respectively).

**Conclusions:**

The incidence of CRPS I among radial head fracture patients undergoing ORIF was 5.5%. In addition, early detection of CRPS I and providing adequate intervention will likely result in greater benefits for those patients.

## 1. Introduction

Radial head fractures are common injuries, accounting for 30% of all elbow injuries and 1.7%–5.4% of all fractures [1]. The radial head plays a crucial role in maintaining the stability of the elbow joint. Therefore, recovering its anatomic location is indispensable for regaining elbow joint function. According to the modified Mason classification, type I (nondisplaced partial fractures) fractures are typically treated nonoperatively [2], whereas surgical management is indicated for types II-IV fractures [1]. The two most frequent surgical procedures are open reduction internal fixation (ORIF) and radial head arthroplasty [3]. Current evidence suggests that the latter is more suitable for comminuted fractures [2], while the former can be employed in most cases. For patients' postoperative function, three elements are considered high priorities: motion, strength, and stability. However, the defining factor for postoperative functional outcome is the ability to perform daily living activities [4].

Complex regional pain syndrome type I (CRPS I) is a postoperative chronic pain condition [5]. A population-based study performed by Sandroni et al. [6] reported that the incidence of CRPS I was 26.2 per 100,000 person-years in Europe. Despite its low incidence, CRPS I may have a significant influence on postoperative function restoration [7]. In CRPS I cases, traumatic events are a significant predisposing factor [8]. Jellad et al. [9] designed a study to specifically investigate CRPS I following distal radius fractures and reported an incidence rate of 32.2% in France. Given that most studies focused on the incidence of CRPS I secondary to distal radius fractures, studies on radial head fractures are limited. This is the first study exploring this topic.

Several studies have reported various risk factors associated with the occurrence of CRPS I in patients with upper extremity fractures, but the results were conflicting [10, 11]. Herein, the variables of interest included psychological conditions, patients' preoperative function, and a few demographic variables. However, the influence of these risk factors in patients with radial head fractures remains unclear. While some studies noted that psychological conditions, such as depression and anxiety, were risk factors for CRPS I [12, 13], others suggested that psychological conditions had no impact on the development of CRPS I [14]. Similarly, some scholars have postulated that patients' preoperative function influences the occurrence of CRPS I, whereas other studies contradicted this statement. Additionally, numerous studies established that CRPS I was correlated with age, gender, and fracture types. However, this observation remains inconsistent, and further investigations are warranted.

We hypothesized that the incidence and associated factors among patients after ORIF would be distinct from other types of fracture. The aim of this prospective observational study was to determine (i) the incidence of CRPS I following ORIF and (ii) the relationship between the risk factors and CRPS I. We believe this article will provide a theoretical foundation for the topic and supplement the existing literature.

## 2. Materials and Methods

### 2.1. Study Design and Setting

This was a cross-sectional study. Patients with radial head fractures treated with ORIF were recruited from the orthopedics department of orthopedic center in China. The study was conducted in accordance with the ethical standards of the 2013 Declaration of Helsinki and approved by the review board of the Third Hospital of Hebei Medical University (2015-002-1). Convenient sampling was used as the sampling method. Informed consent was obtained from all patients prior to initiation of the study. Between January 2017 and March 2020, a total of 946 radial head fracture patients sought care at our institution, and 601 patients who underwent ORIF were enrolled in this study.

### 2.2. Participants

**Figure 1:**
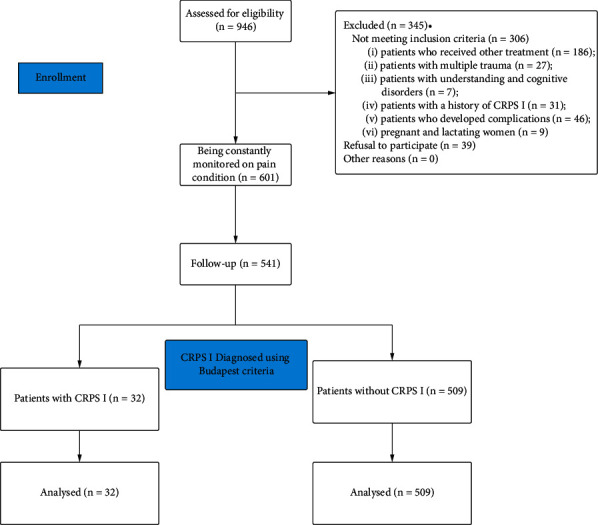
Flow chart of patients included in the present study.

The inclusion criteria were as follows: (1) aged between 18 and 65 years; (2) clinical and radiographic evidence (X-ray, CT scan, and MRI) of radial head fracture and/or associated with ulnar collateral ligament tear, interosseous membrane rupture, and posterior elbow dislocation; (3) patients who underwent ORIF using reduction plates combined with K-wires. The exclusion criteria were as follows: (1) patients who received other treatments, including conservative treatment and radial head prostheses; (2) patients with multiple trauma; (3) patients with understanding and cognitive disorders; (4) patients with a history of CRPS I; (5) patients who developed complications, such as infection and heterotopic ossification, since these complications may increase the incidence of CRPS I; (6) pregnant and lactating women; (7) refusal to participate. The detailed process is illustrated in Figure 1.

### 2.3. Interventions

According to guidelines, all patients received identical treatment, including preoperative education, postoperative rehabilitation training, and pain management [15].

The affected limbs were immobilized with plaster fixation in all patients, and the selective operation was performed within 2 days following admission. The surgery aimed to restore the anatomical structure of the radial head and enhance the stability of soft tissues to allow early mobilization. All treatments were performed by the same surgical team, composed of an anesthesiologist, three orthopedists, and two nurses. Under axillary brachial plexus block and the use of a tourniquet, exposure and fracture reduction were performed through the Kocher approach. The choice of the surgical method depended on the type of fracture and size of bony fragments. Patients with comminuted radial head fractures, where internal fixation was not possible, were excluded from the study. First, the surgical team fixed the fractured radial head using reduction plates combined with K-wires (Zhengtian Medical Technology, Shandong, China). Then, the annular ligament was repaired. Afterward, fracture reduction was examined under a fluoroscope. Finally, the plaster casting was applied for 7 to 10 days after the operation. All patients diagnosed with Mason type IV intraoperatively or through preoperative imaging were excluded from the cohort.

### 2.4. Variables and Data Sources/Measurement

The primary outcome was the incidence of CRPS I. The secondary outcomes were the patients' demographic and clinical characteristics, which were acquired prior to surgery. Data on age, gender, dominant hand, injured side, body mass index, marital status (married, single, widowed, and divorced), educational attainment (university degree, primary and secondary school degrees, and illiterate), job status (employed and unemployed), socioeconomic status (high, middle, and low), type of trauma, history of diabetes mellitus, hypertension and renal disease, and alcohol and tobacco use were recorded. Furthermore, the type of trauma was ranked as high energy (motor vehicle collision/fall from height >1 m), medium energy (fall from <1 m), or low energy (ground-level fall).

The clinical characteristics included fracture classification, pain level, psychological conditions, patients' preoperative function, and quality of life. Fracture classification was determined using the modified Mason classification [1], and the VAS was applied to measure the patients' pain levels. The Hospital Anxiety and Depression Scale (HADS) was employed to assess the patients' psychological conditions (anxiety and depression) [16]. Patient-rated Elbow Evaluation (PREE) [17], and the Quick Disabilities of the Arm, Shoulder, and Hand (QuickDASH) questionnaires [18,19] were employed to evaluate the patients' preoperative upper limb function. Moreover, the Short-Form Health Survey (SF-36) was used to evaluate the patients' quality of life [20].

All patients were followed for at least 12 months, with the primary outcome being the occurrence of CRPS I. At each follow-up visit, a pain specialist measured the patient's pain level using the visual analogue scale (VAS); the pain level cut-off score was established at 50 points [21, 22].

When patients reported disproportionate pain in the operated limb, a screening process was performed. This included routine examinations to exclude potential organic lesions such as infection and nonunion of fractures. If a diagnosis of CRPS II was suspected, nerve conduction studies were performed [12]. As per the Budapest criteria, two out of four clinical signs (sensory, vasomotor, edema, or trophic disturbances) and three out of four symptoms (abnormal sensation, vasomotor changes, edematous changes, or motor dysfunction) are essential for diagnosing CRPS I [23].

### 2.5. Study Size

The primary outcome was the incidence of CRPS I. The sample size of this study was calculated based on the number of variables. The minimum number of included individuals was at least 5–10 times the number of events per variable [24]. Considering four variables were included in this article, the minimum number of individuals in the CRPS I group was 20.

### 2.6. Quantitative Variables

Quantitative variables were expressed as mean and standard deviation for symmetric distribution or median and interquartile range for asymmetric distribution.

### 2.7. Statistical Analysis

The statistical analysis was performed using SPSS 25.0 (SPSS Inc., Chicago, IL, USA). The Shapiro–Wilk test was used as the normality test. For the measurement data meeting the normality assumption (*p* > 0.05), independent *t*-tests were utilized as univariate analyses to compare demographic and clinical data between the CRPS I and control groups. Otherwise, nonparametric tests were used. Categorical variables were examined with either the chi-square test (expected frequency >5) or the Fisher exact test. Herein, independent *t*-tests were employed for comparing age, Quick DASH, SF-36 (physical), and PREE (function and total score) between the two groups. Nonparametric tests were used for BMI, PREE and depression, VAS, SF-36 (mental), depression and anxiety, and PREE (pain). The chi-square test was used to determine the correlation between the incidence of CRPS I and variables such as gender, dominant hand, job status, socioeconomic status, tobacco use, alcohol use, hypertension, diabetes mellitus, and modified Mason Classification. In contrast, the Fisher exact test was used to evaluate the association between the incidence of CRPS I and variables such as the injured side, marital status, educational attainment, type of trauma, and renal disease.

The independent variables were age, gender, dominant hand, injured side, body mass index, marital status, educational attainment, job status, socioeconomic status, type of trauma, history of diabetes mellitus, hypertension and renal disease, alcohol and tobacco use, fracture classification, pain level, psychological conditions, and patients' preoperative function and quality of life. The dependent variable was the occurrence of CRPS I. Multivariate analysis (Backword-Wald) was employed to identify independent factors associated with CRPS I, which included two types of predictors. The first type involved statistically significant predictors (*p* < 0.1) in univariate analyses, and the second type included clinically relevant variables reported in previous studies [25]. The results of the multivariate analyses were expressed as ORs and 95% CIs. 0.05 was used as the cut-off *p* value for statistical significance.

## 3. Results

A total of 523 (87%) out of 601 patients, 98 males and 425 females, completed the 1-year follow-up. The mean time interval from injury to ORIF was 6 days (range of 4–11 days). Based on the Budapest criteria, 28 (5.4%) patients were diagnosed with CRPS I during the first year following surgery. Furthermore, the average time from ORIF to the onset of CRPS I was 12.7 ± 6.2 weeks. Given this was a prospective study, the 10 EPV theory was employed to test the accuracy of the multivariate analysis model. It was revealed that the number of individuals with positive events was 5–10 times the number of variables included in the multivariable logistic regression analysis [24]. When 4 variables were included in the multivariate analysis model, 28 individuals (5.5%) developed positive events (CRPS I), thus conforming to the 10 EPV theory.

The details of the descriptive statistics are listed in Tables 1 and 2. The comparative results between the demographic and clinical characteristics of patients with and without CRPS I are illustrated in Tables 3 and 4. Significant differences were observed in age, gender, modified Mason Classification, and depressive personality disorders. These variables were also regarded as dependent variables and included in the multivariable logistic analysis. In the multivariable logistic analysis, continuous variables were transformed into categorical variables to identify risk factors. Patients were classified as older (>50 years) or younger (≤50 years) adults depending on their age, while their socioeconomic status was determined based on their annual household income: high >12 735, medium = 4126 to 12 735, or low <4126 US Dollar/per year [25]. In addition, the cut-off scores for depression and anxiety were 8 points. Body mass index (BMI) was defined as weight in kilograms divided by height in meters squared (kg/m^2^). The patients were then labeled as underweight (BMI less than 18.5), normal (BMI 18.5–24.9), and obese (BMI more than 24.9).

The results of the multivariable analysis are displayed in Table 5. The factors associated with CRPS I were female gender (OR: 2.633; 95% CI, 1.154–6.008), modified Mason type III fracture (OR: 0.400; 95% CI, 0.181–0.883), and depressive personality disorders (OR: 0.290; 95% CI, 0.098–0.857).

## 4. Discussion

The purpose of this study was to assess CRPS I incidence and identify its associated factors in patients with radial head fractures. According to our results, 5.5% of radial head fracture patients presented with CRPS I symptoms. Female gender, modified Mason type III fractures, and depressive personality disorders were identified as significant predictors of CRPS I. Those findings can aid in the early evaluation and identification of CRPS I in radial head fracture patients.

Radial head fractures account for 17–19% of elbow trauma cases and 33% of elbow fractures [26]. The modified Mason classification is widely used and can provide a reference for the treatment of radial head fractures [27]. Surgical intervention might be more appropriate for modified Mason type II-IV fractures [28]. Indeed, several surgical techniques, such as ORIF, radial head excision, and radial head replacement, have been used in radial head fractures. However, certain approaches such as simple excision often lead to multiple complications such as chronic wrist pain, degenerative arthritis, and so on. Conversely, radial head prosthetic replacement has been regarded as an alternative treatment for severe cases where ORIF is not indicated. Even though ORIF is the most widely preferred surgical technique since it can restore the stability of the elbow joint, preserve the range of motion, and maintain radial length [29], CRPS I can sometimes arise after ORIF. This can lead to poor functional outcomes and dissatisfaction. However, studies focusing on this phenomenon are limited.

Similar to previous findings, the female gender was identified as an independent risk factor for CRPS I. Dutton and Rhee et al. [30] conducted a systematic literature review demonstrating that being female (particularly postmenopausal) was a potential risk factor for CRPS I. It was also concluded that low vitamin C levels partly explained this phenomenon. Nowadays, vitamin C deficiency in females (especially postmenopausal) has become a commonly encountered issue [31]. Additionally, Lucchetta et al. hypothesized that vitamin C might be correlated with postoperative pain relief [32]. Moreover, an earlier study postulated that oral vitamin C supplementation could lower the risk of CRPS I in orthopedic patients [33].

Our study revealed that CRPS I seem more prevalent in modified Mason type III fracture patients. Modified Mason type III fractures represent comminuted fractures with entirely displaced radial heads [1]. The Mason classification divides radial head fractures into 4 groups. As established by previous studies, radial head arthroplasty seems to be more appropriate for modified Mason type IV fractures. Therefore, modified Mason type III fractures are the most severe cases within the specific indication for ORIF. Fracture severity correlates with treatment complexity and future complications, including CRPS I. Therefore, for modified Mason type IV fractures, early focus on diagnosing CRPS I and providing timely intervention is expected to result in greater benefits.

In our study, depressive personality disorder was also considered a risk factor for CRPS I in radial head fracture patients. Hung et al. [34] reported that psychiatric comorbidities (anxiety, depression, etc.) were present in 29% of CRPS patients. Moreover, Duong et al. [35] posited significant associations between depression and CRPS. Nevertheless, anxiety was not identified as a risk factor for CRPS I herein. While some studies reported that psychiatric factors (anxiety and depression) were common comorbidities of CRPS-1 patients, others argued that there was no clear relationship between the two [12, 14]. Since the underlying mechanism behind the relationship between depression and CRPS I remains unknown, further investigations are warranted. Hence, patients' psychological status deserves particular attention when considering CRPS I.

There are some shortcomings in the present study. First, our study only included patients undergoing ORIF. With the lack of a control group, the impact of the surgery on the development of CRPS I could not be determined. It can be inferred that the findings of this study are exclusively applicable to radial head fractures simulated in this work. Second, intraoperative factors such as operative time and intraoperative blood loss were not incorporated in our study, which could lower the accuracy of the model.

## 5. Conclusion

Since the incidence of CRPS I was 5.5% in radial head fracture patients, its early detection and adequate intervention are expected to be highly beneficial for those patients.

## Figures and Tables

**Table 1:** Demographic characteristics of CRPS I Patients.

Characteristics	Sample size	Mean	Standard deviation	95% CI	Minimum	First quartile	Median	Third quartile	Maximum
Age (year)	28.0	41.0	7.6	(38.1, 44.0)	24.0	36.3	40.0	47.0	58.0
The time from fracture to surgery (day)	28.0	5.7	1.2	(5.3, 6.2)	4.0	5.0	6.0	7.0	8.0
Body mass index (kg/m^2^)	28.0	22.6	3.0	(21.4, 23.7)	17.0	21.0	22.0	24.8	30.0
VAS	28.0	17.1	3.3	(15.8, 18.4)	11.0	14.3	17.0	19.8	23.0
QuickDASH	28.0	30.0	7.6	(27.0, 32.9)	17.0	23.6	30.9	36.1	49.0
SF-36 physical	28.0	51.5	3.4	(50.2, 52.8)	45.0	49.0	51.6	54.1	58.0
SF-36 mental	28.0	54.4	5.5	(52.3, 56.5)	46.0	50.0	53.5	58.8	68.0
PREE pain	28.0	32.1	7.2	(29.3, 34.9)	18.0	28.3	32.0	38.3	46.0
PREE function	28.0	25.6	4.7	(23.8, 27.4)	15.0	23.0	25.5	29.8	33.0
PREE	28.0	57.7	9.0	(54.2, 61.2)	42.0	51.0	57.0	64.0	74.0
Anxiety	28.0	7.0	2.2	(6.2, 7.9)	1.0	6.0	7.0	8.0	11.0
Depression	28.0	7.8	1.7	(7.1, 8.4)	6.0	7.0	8.0	12.0	12.0

**Table 2:** Clinical characteristics of study sample.

Characteristics	Patients with CRPS I (*n* = 28)	Patients without CRPS I (*n* = 495)	*p* value
VAS (0–100)	17.7 ± 4.4	17.2 ± 3.1	0.390
Modified Mason classification			
Type II	12	117	
Type III	16	378	0.022^*∗*^
QuickDASH	27.1 ± 6.6	28.4 ± 7.2	0.218
SF-36 (physical)	50.7 ± 3.3	51.4 ± 4.7	0.055
SF-36 (mental)	54.0 ± 2.5	55.2 ± 4.8	0.162
PREE (points)			
Pain (0–50)	33.6 ± 6.1	31.2 ± 7.6	0.081
Function (0–50)	24.2 ± 7.8	25.3 ± 5.4	0.279
Total (0–100)	57.8 ± 13.4	56.5 ± 14.2	0.615
HADS			
Anxiety	6.9 ± 2.3	6.8 ± 2.6	0.832
Depression	7.4 ± 3.1	6.3 ± 0.5	0.001^*∗*^

VAS, 100-mm visual analogue scale; QuickDASH, Quick Disabilities of the Arm, Shoulder, and Hand; PREE, Patient-rated Elbow Evaluation; MEPS, Mayo Elbow Performance Score. Nondominant hand values increased by 5%. ^*∗*^*p* < 0.05.

**Table 3:** Demographic characteristics of study sample.

Characteristics	Patients with CRPS I (*n* = 28)	Patients without CRPS I (*n* = 495)	*p* value
Age (year)	41.0 ± 7.6	49.2 ± 8.2	<0.01^*∗*^
Gender, *n*			
Male	10	88	
Female	18	407	0.018^*∗*^
Dominant hand, *n*			
Left	4	92	
Right	24	403	0.567
Injured side			
Dominant	24	421	
Nondominant	4	74	0.923
Body mass index (kg/m^2^)	22.6 ± 3.0	22.0 ± 2.7	0.263
The time from fracture to surgery	5.7 ± 1.2	5.3 ± 1.7	0.081
Marital status, *n*			
Married	20	284	
Divorced	4	40	
Single	1	109	
Widowed	3	62	0.091
Education, *n*			
University degrees	19	290	
Primary and secondary school degrees	8	161	
Illiterate	1	44	0.108
Job status, *n*			
Unemployed	5	143	
Employed	23	352	0.207
Socioeconomic status, *n*			
High	8	142	
Medium	17	240	
Low	3	113	0.278
Type of trauma			
High energy	20	322	
Medium energy	5	63	
Low energy	3	110	0.322
Tobacco use, *n* (%)	9	137	0.608
Alcohol use, *n* (%)	11	178	0.722
Medical problems			
Hypertension, *n*	13	231	0.980
Diabetes mellitus, *n*	12	151	0.170
Renal disease, *n*	3	34	0.438

**Table 4:** Clinical characteristics of study sample

Characteristics	Patients with CRPS I (*n* = 28)	Patients without CRPS I (*n* = 495)	*p* value
VAS (0–100)	17.1 ± 3.3	17.2 ± 3.3	0.816
Modified Mason classification			
Type II	12	121	
Type III	16	374	0.030^*∗*^
Quick DASH	30.0 ± 7.6	27.9 ± 7.1	0.137
SF-36 (physical)	51.5 ± 3.4	51.5 ± 4.8	0.990
SF-36 (mental)	54.4 ± 5.5	55.3 ± 4.7	0.341
PREE (points)			
Pain (0–50)	32.1 ± 7.2	31.6 ± 7.8	0.738
Function (0–50)	25.6 ± 4.7	25.4 ± 5.2	0.816
Total (0–100)	57.7 ± 9.0	57.0 ± 9.4	0.685
HADS			
Anxiety	7.0 ± 2.2	6.6 ± 2.5	0.418
Depression	7.8 ± 1.7	6.3 ± 2.3	<0.001^*∗*^

VAS, 100-mm visual analogue scale; QuickDASH, Quick Disabilities of the Arm, Shoulder, and Hand; PREE, Patient-rated Elbow Evaluation; MEPS, Mayo Elbow Performance Score. ^*∗*^*p* < 0.05.

**Table 5:** Logistic regression for variables predictive factors of occurrence of CRPS I.

Variable	*β*	Odds ratio	95% CI	*p* value
Female gender	0.968	2.633	1.154-6.008	0.021^*∗*^
Modified Mason III type fracture	−0.917	0.400	0.181-0.883	0.023^*∗*^
Depression personality disorders	−1.238	0.290	0.098-0.857	0.025^*∗*^

Multivariable logistic analysis was used, ^*∗*^*p* < 0.05.

## Data Availability

The datasets used and/or analyzed during the current study are available from the corresponding author upon reasonable request.
